# Cultural adaptation to aging: a study on digital cultural adaptation needs of Chinese older adults based on KANO model

**DOI:** 10.3389/fpubh.2025.1554552

**Published:** 2025-04-02

**Authors:** Sheng Li, Marzudi Md Yunus, Rosila Bee Binti Mohd Hussain, Wenyuan Lin

**Affiliations:** ^1^Institute for Advanced Studies, Universiti Malaya, Kuala Lumpur, Malaysia; ^2^Academy of Malay Studies, Universiti Malaya, Kuala Lumpur, Malaysia; ^3^Department of Anthropology and Sociology, Faculty of Arts and Social Sciences, Universiti Malaya, Kuala Lumpur, Malaysia; ^4^School of Chinese Language and Literature, Beijing Normal University, Beijing, China

**Keywords:** cultural adaptation to aging, KANO model, age-friendly, older adult group, digital cultural adaptation, digital divide

## Abstract

**Introduction:**

With the rapid advancement of digitalization and an aging population, China faces unprecedented challenges in older adults’ digital cultural adaptation. The “54th Statistical Report on China’s Internet Development” reveals that individuals aged 60 and above constitute 14.3% of total internet users. However, significant barriers persist in their digital participation, necessitating a deeper understanding of their adaptation process and needs.

**Methods:**

This study employs the KANO model to assess older adults’ digital cultural adaptation. A total of 205 respondents participated in a questionnaire survey, with data analyzed using the highest frequency method and the Better-Worse coefficient. The study categorizes digital cultural needs into three dimensions: must-be requirements, one-dimensional requirements, and attractive requirements.

**Results:**

The findings indicate that: 1. Older adults’ digital cultural needs comprise must-be requirements (basic functional design, 47.8%), one-dimensional requirements (social participation, 36.1%), and attractive requirements (innovative experiences, 66.8%). 2. Intergenerational cultural cognitive differences, digital skill levels, and the urban–rural digital divide significantly impact their adaptation process. 3. While respondents positively evaluate digital life satisfaction, personal adaptation, and digital content diversity, they express dissatisfaction and expectations regarding family guidance, age-friendly digital services, and the role of digital content in improving life quality.

**Discussion:**

The results highlight the need for targeted strategies in technical support, content design, and service provision to enhance older adults’ digital cultural adaptation. Addressing group-specific needs and preferences through coordinated measures can improve digital inclusion, foster intergenerational cultural integration, and optimize digital cultural service systems. This study offers valuable theoretical insights and practical implications for advancing policies and initiatives that support older adults in the digital era.

## 1 Introduction

China is experiencing the world’s largest and fastest population aging process. According to the United Nations Department of Economic and Social Affairs, the global population aged 65 and above is projected to increase from 10% in 2022 to 16% by 2050 (1). China’s aging trend is even more pronounced, with the “54th Statistical Report on China’s Internet Development” showing that internet users aged 60 and above account for 14.3% of total users (2).

As digitalization and population aging emerge as two distinctive features of contemporary Chinese social transformation (3), promoting digital inclusion for older adults and ensuring their equal access to digital benefits has become a focal point for both government and society. Digital technology significantly empowers the older adult population, enhancing both their physical and mental health while promoting social participation (4). Particularly in digital cultural services, technology can provide older adults with richer cultural content, more convenient service access channels, and broader social interaction opportunities. However, a considerable proportion of older adults currently face an insurmountable digital divide (5). This divide not only prevents them from enjoying digital benefits but also partially excludes them from normal social functioning, leading to social isolation and digital exclusion. Research indicates that older adults’ digital divide shows progressive characteristics of “digital access divide, capability divide, and outcome divide” (6). Meanwhile, significant regional and urban–rural digital disparities exist among China’s older adult population: urban internet users account for 72.1% of total users (2), with rural older adults facing a more severe digital divide and weaker capacity to cope with digitalization compared to their urban counterparts (7). These disparities are manifested across multiple dimensions, including digital infrastructure accessibility, socioeconomic status, education level, and social support.

Existing research shows that older adults exhibit unique characteristics in digital technology usage. They prefer smartphones over devices like iPads and computers due to easier operation (8). Functionally, they primarily focus on communication and entertainment features, while showing lower usage frequencies of advanced functions like online shopping and mobile payments. These phenomena indicate that older adults’ digital participation remains at a preliminary stage, with their participation willingness and behavior influenced by multiple factors (7).

While a rich body of literature has accumulated on older adults’ digital participation, existing research in the specific field of digital cultural services has primarily focused on technical aspects (such as interface design and functional configuration), while systematic research on digital cultural content remains relatively limited. In particular, studies using the KANO model to classify and prioritize digital cultural adaptation needs of Chinese older adults are notably scarce. Moreover, most existing research is predominantly based on Western developed countries, and the unique challenges and needs faced by Chinese older adults in digital cultural adaptation have not been fully explored. Through the innovative application of the KANO model, this study not only systematically analyzes the hierarchical characteristics of Chinese older adults’ digital cultural needs, but also provides quantitative assessment of need priorities through Better-Worse coefficient analysis, offering theoretical foundation and practical guidance for formulating policies to promote digital inclusive development for older adults.

## 2 Literature review

### 2.1 Cultural adaptation to aging

Older adults’ cultural construction is an essential component of the cultural power strategy. With the accelerating development of an aging society, age-appropriate research and design have gained increasing attention (9). However, existing literature lacks systematic definition and research exploration of the concept “Cultural Adaptation to Aging” (10).

The “aging” in Cultural Adaptation to Aging refers not only to the older adult population but also encompasses traditional excellent cultural clusters (10). In the development of contemporary spiritual and trend culture, it is essential to maintain cultural origins, achieving dynamic inheritance, constraint, development, and integration based on traditional cultural inheritance. Particularly under China’s “14th Five-Year Plan” promoting cultural industry digitalization, traditional culture needs to adapt to contemporary social, technological, and cultural development to better achieve cultural promotion and inheritance, advancing the strategic goal of building a cultural power.

Cultural adaptation refers to the adjustment of interventions to better accommodate individuals’ language, cultural values, and norms (11). The objective is to achieve inclusivity, ensuring that all individuals and communities, including the most vulnerable groups, can access and utilize technology to enhance their quality of life (12). There are different models to guide the cultural adaptation of interventions. The most commonly used models are the Cultural Adaptation Process (CAP) model and the Ecological Validity Model (EVM) (13, 14). Beyond economic and physical factors, disparities in access to and use of digital health interventions are culturally driven. Creating inclusive digital health interventions is crucial to avoid systematically excluding traditionally underserved cultural groups (13). The fundamental rationale behind cultural adaptation is to improve coverage and engagement among otherwise underserved population subgroups (15, 16).

In studying the cultural adaptation of digital health interventions, there exists a complex interplay among three dimensions: culture, technology, and health. Cultural beliefs and values profoundly influence people’s health perceptions and behaviors, while the introduction of digital technology transforms traditional healthcare delivery and health management approaches. Meanwhile, cultural factors determine the degree of technology adoption and usage patterns, and conversely, the application of technology may either facilitate or hinder health practices among specific cultural groups. Understanding this multidimensional interaction is crucial for developing effective digital health interventions. Although designing culturally sensitive digital health interventions from scratch might achieve optimal cultural fit, it requires substantial resources. In comparison, culturally adapting existing technologies, while still requiring time and financial investment, often presents a more feasible solution. Successful digital health interventions must simultaneously consider technological functionality, cultural adaptability, and health outcomes. This multidimensional bidirectional interaction demands a holistic approach that views cultural adaptation as an ongoing dialogue between technological capabilities and cultural needs (13).

### 2.2 Research on digital cultural adaptation to aging

Digital technology has significant empowering effects on older adults: it provides basic functions like healthcare and banking services, and promotes social connections and daily activity participation, thereby enhancing older adults’ physical and mental health and social interaction (17, 18). Taking mobile payment as an example, its positive impact on older adults’ well-being is mainly reflected in providing life convenience, optimizing consumption structure, increasing social interaction opportunities, and improving overall quality of life (18). Research indicates that the digital divide among older adults may exacerbate health disparities and social isolation (19). Tian& Li further found that Internet use plays an important moderating role in the relationship between social networks and mental health in older adults (20). Their study of 7,648 Chinese adults over 60 years old showed that Internet use exerts different moderating functions in the impact of family networks on loneliness and friend networks on mental health, highlighting the importance of utilizing Internet technology to protect the well-being of older adults in a digital society.

Current research on digital cultural adaptation to aging primarily focuses on technical aspects, such as interface design and functional configuration, including font size, color contrast, operation process simplification, and intelligent assistance functions. However, content-level research remains insufficient, lacking corresponding studies. This research status reflects both the limitations of technology-oriented thinking and highlights the necessity of conducting content-level research from user experience and service design perspectives. Digital technology significantly impacts older adult care system construction and older adults’ health and can extend to education, culture, tourism, and other fields, indicating its potential to further meet older adults’ spiritual and cultural needs. Beyond meeting basic life needs, reasonable application of digital technology will effectively enhance older adult group’s happiness and sense of achievement (7). Digital technology is not limited to mobile payments; smart homes, as the new generation of essential terminals for future homes, have also become an important medium for intelligent older adult care at home. Related research shows that the main factors affecting older adults’ use of smart home social media include user interface quality, interaction quality, content quality, and service quality. These factors influence older adults’ emotional experiences and perceptions through social compensation mechanisms, thereby affecting their willingness to accept and use smart home social media (21).

### 2.3 Digital cultural services for Chinese older adults

In recent years, China has highly valued older adults’ cultural service needs. The “Opinion on Promoting High-Quality Development of Public Cultural Services” jointly issued by 16 departments, including the National Office on Aging, clearly proposes providing more high-quality, suitable cultural products and services for older adults (10). Meanwhile, to address deepening population aging, departments including the Ministry of Industry and Information Technology have issued action plans for smart older adult care industry development, focusing on developing intelligent products and information systems, bringing new opportunities for social older adult care systems and older adults’ cultural development. To meet diverse older adults’ needs, age-appropriate design should not be limited to solving basic survival needs but should focus on exploring “silver economy” consumption potential and improving service quality in spiritual aspects such as emotional communication and social recognition (22).

**Figure 1:**
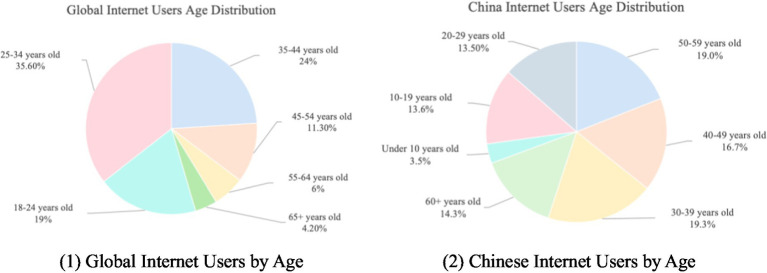
Distribution of internet users worldwide as of February 2024 by age group. (1) Global internet users by age (2) Chinese internet users by age.

The pie chart in Figure 1 (1) depicts global internet users by age group (23), with users aged 65 and above accounting for 4.2%. Figure 1 (2) depicts Chinese internet users by age group, with users aged 60 and above accounting for 14.3% (2).

Despite differences in age classification standards, this data indirectly reflects the relatively high internet penetration rate among Chinese older adult groups, indicating China’s significant achievements in promoting digitalization among the older adult population. This high penetration rate may be closely related to China’s aging population trends and digital inclusion policies.

Currently, although digital inclusion has become an essential component of active aging and Digital China strategy, existing policies mainly focus on age-friendly modifications, digital resource provision, and technology promotion, with insufficient attention to cultural services and spiritual needs (24). Research indicates that both social and digital inclusion are significantly influenced by institutional environment and macro policies, suggesting the need for greater policy-level emphasis on older adults’ cultural service needs (7).

**Figure 2:**
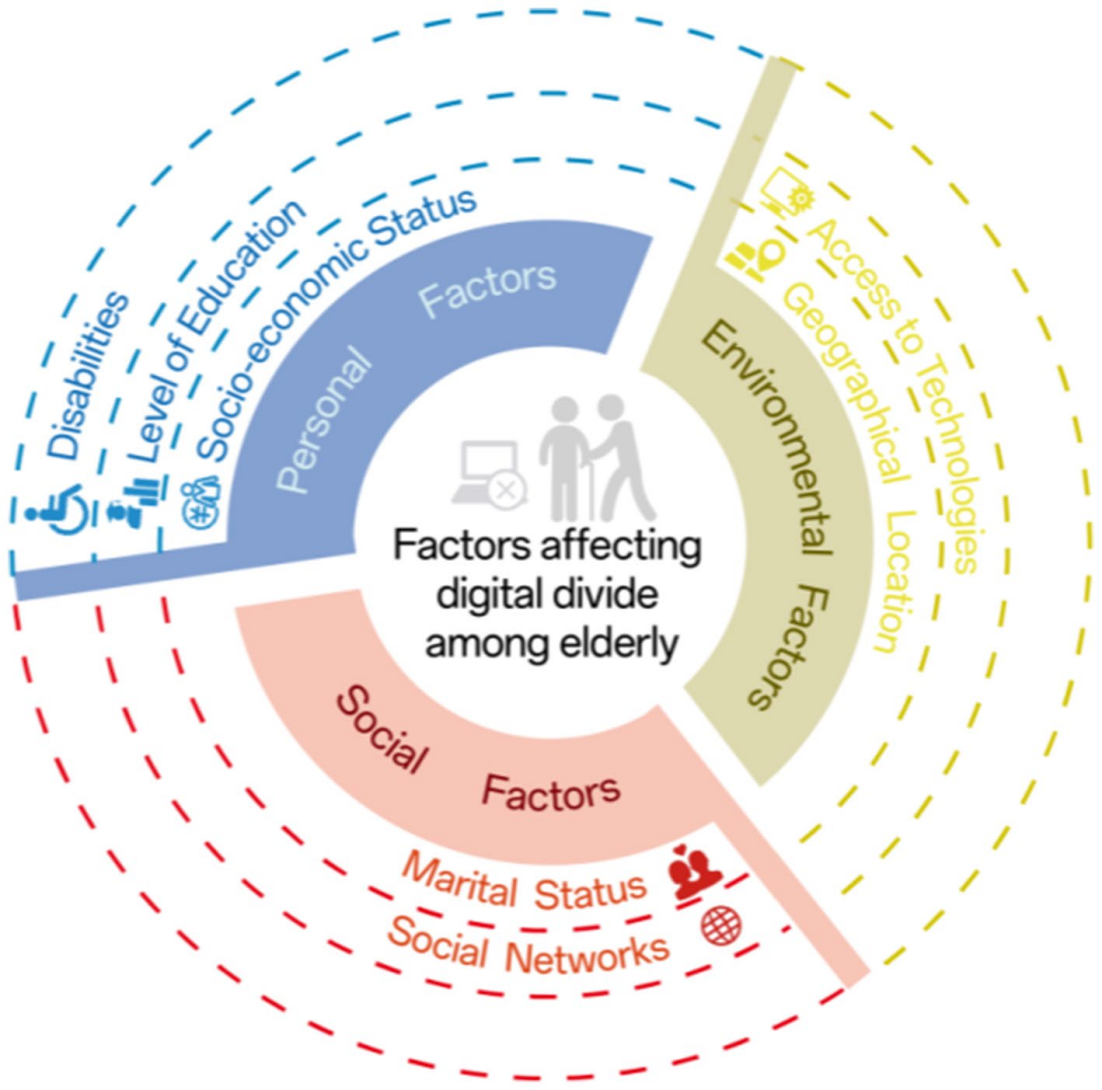
Three dimensional indicators of digital exclusion among older adults.

Research on older adults’ media literacy reveals a significant digital divide between older adults and young people. Studies show that older adults’ digital literacy, such as internet information retrieval ability, is significantly lower than younger groups (with a 17% difference) (25). According to Lythreatis et al. (26), causes of older adults’ digital exclusion can be categorized into personal, social, and environmental dimensions (5), with specific factors detailed in Figure 2 (9).

Substantial research evidence demonstrates that internet penetration has profoundly changed people’s thinking patterns and behavioral modes (27, 28). Particularly in the aging process, older adult groups gradually participate in building an inclusive digital society through active use of digital technologies like smartphones (29).

However, while existing research on the digital divide primarily focuses on Western developed countries such as the UK, Canada, and Switzerland (30, 31), studies addressing the “grey digital divide” faced by Chinese older adult groups have increased in recent years.

### 2.4 Digital aging-related policies

**Figure 3:**
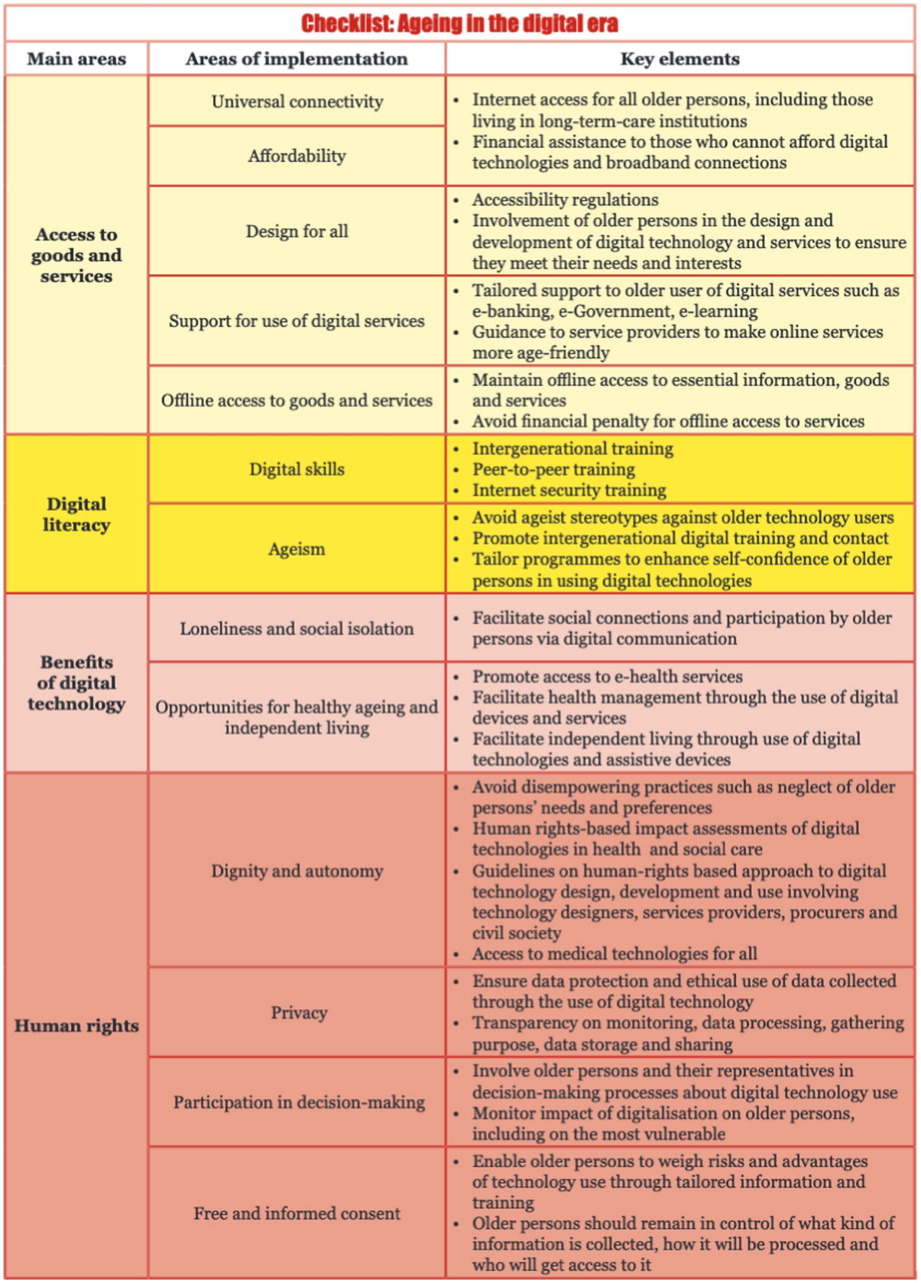
Checklist: UNECE policy brief on aging no. 26.

To address these issues, policy strategies need to encompass aspects of enhancing digital inclusion and empowerment of older adults. In some countries, established practices of incorporating older adults in digital enhancement can be adapted or replicated in other contexts. ECE member states have developed a range of policies promoting older adults’ active participation in digital research, development, and decision-making, as illustrated in Figure 3. The valuable life experiences and diverse perspectives of older adults are crucial for bridging the digital divide and contributing to a world for all ages (32).

## 3 Research methods and data analysis

### 3.1 Theoretical foundation of KANO model

**Figure 4:**
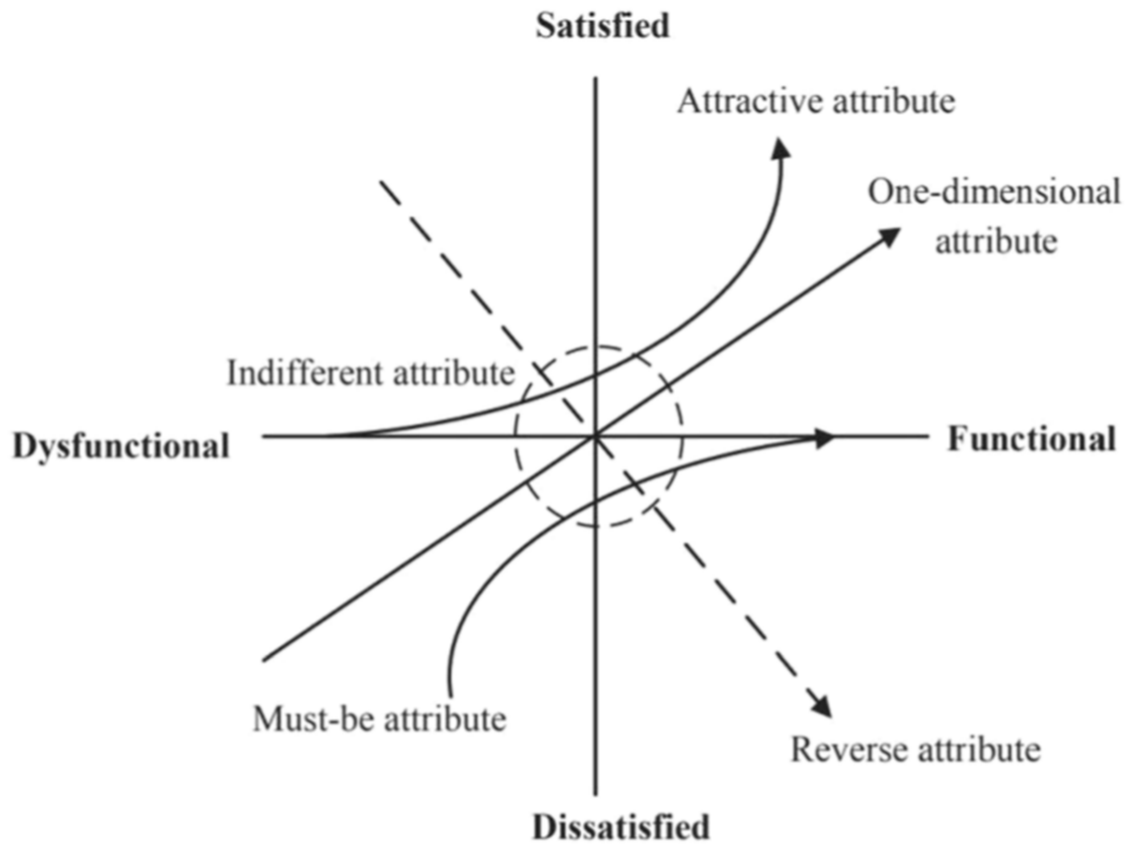
KANO model quality attribute classification diagram (52).

The KANO model, proposed by Japanese scholar KANO (33), is a user requirement analysis method based on user satisfaction and quality function availability, primarily used for user requirement classification and priority ordering. The KANO model categorizes user requirements into five types: Must-be (M), One-dimensional (O), Attractive (A), Indifferent (I), and Reverse (R) requirements (see Figure 4 and Table 1). In practical applications, the KANO model evaluates user requirement characteristics through its unique attribute matrix. The evaluation method employs the highest frequency method, determining the final attribute category by calculating the frequency of each requirement attribute type.

**Table 1:** KANO model highest frequency method evaluation result classification reference.

Feature/service		Negative question				
		Disgusting (1 points)	Reluctant (2 points)	Indifferent (3 points)	Necessary (4 points)	Favorite (5 points)
Positive question	**Disgusting** (1 points)	Q	R	R	R	R
	**Reluctant** (2 points)	M	I	I	I	R
	**Indifferent** (3 points)	M	I	I	I	R
	**Necessary** (4 points)	M	I	I	I	R
	**Favorite** (5 points)	O	A	A	A	Q

A, Attractive; O, One-dimensional; M, Must-be; I, Indifferent; R, Reverse; Q, Questionable.

### 3.2 Theoretical framework

This study explores Chinese older adults’ digital cultural adaptation needs using mixed research methods based on the KANO model theoretical framework. The theoretical basis for selecting the KANO model includes:

Scientific Quality Attribute Classification: The KANO model systematically categorizes user requirements into five types: Must-be (M), One-dimensional (O), Attractive (A), Indifferent (I), Reverse (R) and Questionable (Q), helping comprehensively grasp older adult users’ requirement characteristics.

Dual-dimensional Satisfaction Assessment: Through unique evaluation methods pairing positive and negative questions, it examines both the positive impact of an attribute’s presence on satisfaction and the negative impact of its absence on dissatisfaction.

Objective Priority Judgment: The Better-Worse coefficient analysis method provides quantitative evaluation standards, objectively determining requirement priority levels.

### 3.3 Analysis of older adult user needs based on KANO model

The KANO model has been widely applied in analyzing older adult group design user needs, particularly in age-appropriate research. For instance, Shi, Chongqing et al. analyzed the importance and influencing factors of social support service needs for urban older adults with dementia using the KANO model, employing Better-Worse coefficient and sensitivity formula methods (34). Huang Guoliang et al. applied the KANO model to determine attribute categories and user value importance of 12 functional programs in intelligent companion robots (35). While Xinxin Sun et al.’s research used Behavioral Reasoning Theory (BRT) as a framework, combined with the UTAUT2 model, and found that values significantly influence older adults’ attitudes toward using companion robots (36). Chengmin Zhou et al. explored older adult individual self-care needs design requirements and user pain points using the KANO model, summarizing functional requirements for older adult-friendly digital service platforms and prioritizing key needs (37). Additionally, research has applied this model to enhance user satisfaction (38).

In age-appropriate research of digital interactive devices, the KANO model has been extensively applied to user needs in medical and health applications (39), age-appropriate reading APP design requirements (40), customized tourism APP design (41), and smart watch interaction design (42). However, research in digital cultural content remains relatively limited. While some scholars have used KANO theory to explore older adults’ personalized game element selection (43) and older adult group participation in self-media needs and services (44), these studies only address older adults’ needs and usage benefits regarding digital cultural content, showing insufficient systematic research and analysis of older adult group adaptation to new digital cultural content (Table 2).

**Table 2:** KANO questionnaire example: older adults’ digital media usage needs assessment.

A1: If digital media platforms have special designs for older adults (large font, simple layout, important news marking)					
For having this feature/attribute, I feel:	Favorite	Necessary	Indifferent	Reluctant	Disgusting
For not having this feature/attribute, I feel:	Favorite	Necessary	Indifferent	Reluctant	Disgusting

### 3.4 KANO questionnaire design

Based on KANO model construction principles, dimensions were confirmed through systematic literature review to customize both positive and negative questions for investigating Chinese older adult group’s digital cultural adaptation. Each question provides 5 response options: “Disgusting,” “Reluctant,” “Indifferent,” “Necessary,” and “Favorite.” Respondents select the most appropriate answer based on their feelings and understanding to determine requirement attributes (Table 3).

**Table 3:** Indicator system for older adults’ digital cultural adaptation needs based on KANO model.

Code	Need indicators
A1	If digital media platforms have special designs for older adults (large font, simple layout, important news marking)
A2	If social media (like WeChat) has dedicated older adult mode
A3	If able to understand and explain new internet expressions (daily phrases, greetings, compliments, trending terms)
A4	If able to understand and participate in current digital culture (short videos, live streaming, online communities)
A5	If older adult-appropriate digital learning methods are available (text, video, audio, interactive, games)
A6	If easy access to interesting digital content (health, culture, news, lifestyle)
A7	If digital platforms automatically simplify functions based on usage habits
A8	If able to understand young people’s lifestyle and values through digital means
A9	If no digital divide impact in intergenerational communication
A10	If easy adaptation to digital lifestyle (online shopping, mobile payment, online appointments)
A11	If digital platforms recommend suitable entertainment and learning content
A12	If able to build rich social connections in digital world (reunions, interest groups, neighborhood interaction)
A13	If able to participate in digital cultural entertainment (viewing, interaction, creation, sharing)
A14	If able to participate in community activities, volunteer services, cultural events through digital channels
A15	If able to enjoy diverse digital entertainment (live streaming, short videos, online karaoke, puzzle games)
A16	If able to inherit and promote traditional cultural values through digital means
A17	If digital platforms make frequently used functions one-click operations
A18	If digital platform interface design considers older adults’ visual needs (color contrast, button size)
A19	If all digital platform functions have voice operation options
A20	If digital product appearance has good aesthetic design
A21	If function format and operation convenience are well matched
A22	If able to accept new things while maintaining traditional values
A23	If able to distinguish authentic online information and avoid scams
A24	If understand cybersecurity knowledge to protect personal privacy and property
A25	If able to master basic digital platform usage skills
A26	If able to naturally express emotions in digital world (text, voice, emoticons, gifts)
A27	If able to share life stories, knowledge, and talents through digital means
A28	If digital cultural participation promotes personal growth
A29	If digital culture enables better integration into modern society
A30	If able to access personalized digital cultural experiences

To ensure accuracy and validity, the draft questionnaire underwent pilot testing, followed by detailed revision and optimization. The final questionnaire consists of:

- Demographic information (4 items)
- Digital device usage (5 items)
- KANO model evaluation items (30 pairs of positive–negative questions)

The KANO model-based questionnaire comprises paired questions. For each indicator, the survey includes both positive and negative questions using a 5-point scale.

### 3.5 Questionnaire collection

This study conducted questionnaire distribution and collection through online channels. The questionnaires were distributed through WeChat community groups for older adults, online platforms of universities for older adults, and existing network channels of community activity centers for older adults, using the professional platform WJX.cn for questionnaire design and data collection. The research established clear screening criteria: respondents must be 60 years or older (conforming to China’s official definition of older adults), and have basic experience using digital devices (questionnaires could be completed with assistance from children or family members).

A total of 245 questionnaires were collected, and after data cleaning and validation (excluding incomplete responses and questionnaires with obvious logical contradictions), 205 valid responses were obtained, resulting in an effectiveness rate of 83.7%. Each questionnaire took approximately 3–5 min to complete. All collected data, including responses to both positive and negative questions, were imported into SPSS software for analysis. The research chose to distribute questionnaires during concentrated periods of community older adults’ activities; this concentrated data collection strategy helped reduce potential bias from time differences, providing a reliable data foundation for subsequent statistical analysis.

### 3.6 Questionnaire reliability

Reliability testing examines the questionnaire results’ reliability, stability, and consistency, reflecting whether measurement results can truthfully represent respondents’ consistent and stable characteristics. A higher reliability coefficient indicates better representation of respondents’ consistent and stable characteristics. The Cronbach’s α coefficient is the most commonly used reliability coefficient, evaluating the consistency of scores across questionnaire items. It is an internal consistency reliability coefficient, where k represents the number of survey items, $S_{i}^{2}$ is the variance of the i-th item score, and $S_{T}^{2}$ is the total score variance (Table 4).


(1) $$\alpha =\frac{k}{k-1}\left(1-\frac{\sum_{i=1}^{k}S_{i}^{2}}{S_{T}^{2}}\right)$$

**Table 4:** Cronbach’s α coefficient reliability test results.

(1) Positive questions reliability analysis		
Items	Sample size	Cronbach’s α
30	205	0.956
The positive questions’ reliability coefficient of 0.956 exceeds 0.9, indicating very high data reliability		

(2) Negative questions reliability analysis		
Items	Sample size	Cronbach’s α
30	205	0.957
The negative questions’ reliability coefficient of 0.957 exceeds 0.9, indicating very high data reliability.		

### 3.7 Validity testing

Validity testing examines questionnaire effectiveness, measuring how accurately the tool measures the intended subject. Higher correspondence between test results and research questions indicates higher validity. Structural validity refers to the degree of correspondence between questionnaire structure and measurement values (Table 5).

**Table 5:** KMO and Bartlett’s sphericity test results.

KMO value		0.952
Bartlett’s test of sphericity	Approx. Chi-Square	10712.826
	*df*	1770
	*p*-value	0.000

The KMO value of 0.952 (>0.8) indicates the research data is highly suitable for information extraction, confirming the data passes validity testing.

## 4 Results analysis

### 4.1 Basic situation analysis

This study collected data across three dimensions to understand older adults’ digital cultural adaptation needs: Demographic characteristics, Usage behavior and Barriers and needs. Through analyzing basic characteristics and digital device usage patterns across different groups, the study reveals the diversity of older adults’ digital cultural needs and their main influencing factors (Table 6).

**Table 6:** Survey respondent demographic distribution statistics.

Categories	Options	Frequency	Percentage (%)
Age	60–65 years old	26	12.68
	66–70 years old	78	38.05
	71–75 years old	56	27.32
	76–80 years old	26	12.68
	Over 80 years old	19	9.27
Education level	Primary school or below	65	31.71
	Middle school	95	46.34
	High school/Technical	31	15.12
	College or above	14	6.83
Residence area	Township/City	118	57.56
	Rural	87	42.44
Living situation	Living alone	55	26.83
	Living with family	137	66.83
	Other	13	6.34
Current health status	Completely healthy	63	30.73
	Minor discomfort	93	45.37
	Chronic illness	31	15.12
	Limited mobility	18	8.78
Family meeting frequency	Daily	137	66.83
	Several times/week	26	12.68
	Several times/month	35	17.07
	Rarely	7	3.41
Daily smart device usage	Under 1 h	10	4.88
	1–3 h	97	47.32
	3–5 h	71	34.63
	Over 5 h	27	13.17
Digital device usage difficulty	No difficulties	119	58.05
	Some difficulties	63	30.73
	Significant difficulties	23	11.22
Frequency of asking young people for help	Never	20	9.76
	Rarely (1–2/month)	44	21.46
	Occasionally (1–2/week)	89	43.41
	Often (3–5/week)	29	14.15
	Frequently (daily)	23	11.22
Each total		205	100

The survey respondents were predominantly aged 66–70 (38.05%), indicating younger older adults are the main digital device users. Education levels were mainly middle school and below (78.05%), with slightly higher urban township representation compared to rural areas (57.56% vs. 42.44%).

Regarding usage behavior:

- Smartphones are the primary digital device (88.78%)
- Most use devices 1–3 h daily (47.32%)
- Main purposes: family communication (87.32%) and entertainment (78.54%)
- 43.41% seek weekly guidance from family members on device usage

Overall, while respondents show interest in digital cultural adaptation, they face limitations from education levels, urban–rural disparities, and physical conditions. Future digital service design should account for these differences to promote broader older adults’ digital inclusion.

### 4.2 Satisfaction assessment of digital cultural adaptation among the older adult population

Based on the demographic characteristics and usage behaviors mentioned above, this study further investigated the satisfaction and evaluation of the older adult group in the process of digital culture adaptation. By analyzing the feedback of respondents in different dimensions, their actual needs can be understood.

From the five options of very dissatisfied (1 point), somewhat dissatisfied (2 points), average (3 points), basically satisfied (4 points), and very satisfied (5 points), the table below presents the statistical results of ratings in several key areas, including mean, standard deviation, skewness coefficient, and kurtosis coefficient. Among them, the mean reflects the overall evaluation tendency of the respondents, while the standard deviation reflects the degree of dispersion of individual ratings. The skewness and kurtosis coefficients are used to assess the deviation of the data from the normal distribution (Table 7).

**Table 7:** Descriptive statistical results of satisfaction with digital culture adaptation among the older adult population.

Evaluation content	Average value	Standard deviation
Your current satisfaction with digital life	4.327	1.178
Your satisfaction with your own level of adaptation	2.054	1.489
Your satisfaction with the guidance from your family	4.302	1.199
Your satisfaction with the age-appropriateness of digital services	2.922	1.122
What is your evaluation of the richness and diversity of digital content in the new era, such as online education, health consultation, entertainment activities, etc	4.356	1.127
How much do you think digital content in the new era can help improve your quality of life	2.029	1.441

Overall, the older adult group’s satisfaction and evaluation of digital cultural life present some noteworthy characteristics. In terms of overall satisfaction with digital life and their own adaptability, most older people hold a positive attitude, with average scores close to full marks and small individual differences. This indicates that with the continuous development of digitalization, the older adult group has gradually realized the importance of integrating into the digital society and made efforts to improve their own digital literacy.

However, in terms of family guidance and the age-appropriateness of digital services, the satisfaction of older adults is generally low, with large differences in personal evaluations. This reflects that the current digital environment still has deficiencies in its friendliness and inclusiveness toward the older adult group. Especially in terms of family support, more attention needs to be paid to strengthening intergenerational communication and interaction. Regarding digital content in the new era, older adults generally recognize its richness, but have doubts about its actual effectiveness in improving their quality of life. This suggests that when providing content, we should focus more on pertinence and emotional value, rather than just focusing on the amount of information.

From the data distribution, most scores are concentrated around the mean, but there are also certain extreme values. Satisfaction and content evaluation are generally high, while scores for family guidance and quality of life improvement are relatively low.

In summary, respondents hold positive evaluations in terms of digital life satisfaction, their own level of adaptation, and the richness and diversity of digital content in the new era. However, there is dissatisfaction and expectation in terms of satisfaction with family guidance, the age-appropriateness of digital services, and the degree to which digital content in the new era helps improve quality of life. This feedback provides valuable reference information for relevant parties and helps to further improve and optimize digital services and enhance overall user satisfaction. At the same time, the non-normal distribution characteristics of the data also remind us that when interpreting statistical results, we need to comprehensively consider the differentiated demands of different groups, formulate targeted service strategies and communication plans, and promote the overall improvement of digital literacy and cultural adaptation capabilities of older adults.

### 4.3 KANO quality survey

The research conducted in-depth analysis of questionnaire data using the KANO model to classify older adults’ digital cultural service needs. The KANO model categorizes user requirements into five types: Must-be, One-dimensional, Attractive, Indifferent, and Reverse, helping clarify positive and negative impacts of different needs on satisfaction. This section summarizes KANO model analysis results and evaluates need priorities using the Better-Worse coefficient method (see Table 8) for detailed results.

**Table 8:** Summary of KANO model analysis results.

Functions/services	A	O	M	I	R	Q	Result
A1	10.24%	8.29%	47.80%	23.90%	9.76%	0.00%	M
A2	11.71%	9.76%	54.15%	14.63%	9.76%	0.00%	M
A3	18.05%	11.71%	19.51%	31.71%	19.02%	0.00%	I
A4	39.02%	12.20%	14.15%	25.37%	9.27%	0.00%	A
A5	17.07%	9.76%	44.88%	20.00%	8.29%	0.00%	M
A6	20.49%	17.56%	11.22%	37.07%	13.66%	0.00%	I
A7	40.00%	10.24%	13.17%	24.39%	12.20%	0.00%	A
A8	56.59%	5.37%	9.27%	22.44%	6.34%	0.00%	A
A9	20.49%	13.17%	13.66%	37.56%	15.12%	0.00%	I
A10	11.22%	6.34%	57.07%	17.56%	7.80%	0.00%	M
A11	5.85%	11.71%	45.85%	26.34%	10.24%	0.00%	M
A12	19.02%	15.61%	19.02%	30.73%	15.61%	0.00%	I
A13	43.41%	11.71%	10.24%	25.37%	9.27%	0.00%	A
A14	13.17%	36.10%	12.68%	25.85%	12.20%	0.00%	O
A15	66.83%	6.83%	6.83%	15.12%	4.39%	0.00%	A
A16	13.17%	32.68%	12.68%	30.24%	11.22%	0.00%	O
A17	39.51%	11.22%	13.66%	25.37%	10.24%	0.00%	A
A18	15.61%	12.68%	20.49%	35.12%	16.10%	0.00%	I
A19	65.37%	5.85%	10.24%	11.22%	7.32%	0.00%	A
A20	13.17%	9.76%	46.83%	20.98%	9.27%	0.00%	M
A21	10.73%	9.27%	47.32%	22.44%	10.24%	0.00%	M
A22	15.61%	16.59%	18.54%	32.20%	17.07%	0.00%	I
A23	65.85%	6.34%	7.80%	14.63%	5.37%	0.00%	A
A24	67.32%	6.83%	4.39%	15.61%	5.85%	0.00%	A
A25	12.68%	50.73%	9.76%	20.49%	6.34%	0.00%	O
A26	66.34%	3.41%	7.80%	16.10%	6.34%	0.00%	A
A27	12.68%	9.27%	43.90%	24.88%	9.27%	0.00%	M
A28	11.22%	52.20%	12.68%	14.63%	9.27%	0.00%	O
A29	71.71%	3.90%	4.88%	12.20%	7.32%	0.00%	A
A30	52.68%	7.32%	9.76%	22.93%	7.32%	0.00%	A

A, Attractive; O, One-dimensional; M, Must-be; I, Indifferent; R, Reverse; Q, Questionable.

According to the KANO model analysis results, older adults’ digital cultural service needs can be clearly divided into four types: Must-be attributes (M), One-dimensional attributes (O), Attractive attributes (A), and Indifferent attributes (I). Through in-depth analysis of these attributes, we can better understand the core demands and experience preferences of older adult groups in their digital cultural adaptation process.

Must-be attributes: These attributes are basic guarantees provided by digital media platforms for older adults. Large fonts, simple layouts, and important news marking ensure older adults can easily read and understand information. Dedicated older adult modes and age-appropriate digital skill learning methods help them better adapt to and use digital products. Additionally, platforms should support older adults in easily adapting to digital lifestyles, such as online shopping and mobile payments, and recommend suitable entertainment and learning content. Meanwhile, product appearance design and functional form should match operational convenience, allowing older adults to conveniently share their life stories, knowledge, and talents.

Attractive attributes: These attributes help older adults maintain vitality and engagement in the digital world. Understanding and participating in current popular culture, such as short videos and livestreaming, helps them maintain communication with young people and understand their lifestyles and values. Rich digital cultural entertainment activities and entertainment methods provide more choices and enjoyment. One-click and voice operation options simplify operation processes, improving older adults’ user experience. Platforms should also help older adults distinguish authentic online information and protect personal privacy and property security, making them more confident and secure in the digital world.

One-dimensional attributes: These attributes emphasize older adults’ social participation and personal growth in the digital world. Participating in community activities and volunteer services through digital channels helps older adults integrate into society and contribute their experience. Inheriting and promoting traditional cultural values helps older adults maintain cultural roots in the digital world. Mastering basic digital platform usage skills is fundamental for older adults’ independent use of digital products. Digital cultural participation can also promote older adults’ personal growth and development, improving their quality of life.

Indifferent attributes: While these attributes have less impact on older adults’ digital experience, they remain important. Timely understanding and explanation of new internet expressions helps older adults overcome language barriers and better communicate with young people. Easy access to interesting digital content meets older adults’ information needs. Building rich social relationships in the digital world helps older adults maintain social activity and reduce loneliness. Interface design considering older adults’ visual needs improves their user experience. Meanwhile, accepting new things while maintaining traditional values is key for older adults to maintain balance and harmony in the digital world.

In conclusion, these four attributes collectively form essential components of older adults’ digital experience, each indispensable, jointly creating a friendly, convenient, rich, and secure digital world for older adults.

### 4.4 Better and worse coefficient analysis results

Addressing potential inaccuracies in relying solely on the highest frequency method for classifying user requirement attributes, and difficulties in reflecting the impact of requirement elements on overall satisfaction, Charles Berger et al. proposed the Better-Worse coefficient analysis method as an improvement (45). This method applies statistical data of various requirement attribute frequencies to calculate Better coefficients (satisfaction coefficients, typically positive) and Worse coefficients (dissatisfaction coefficients, typically negative). The Better-Worse coefficient analysis method is a tool for evaluating how requirement fulfillment affects overall satisfaction differences. The Better coefficient reflects how much a requirement’s fulfillment contributes to improving overall satisfaction, ranging from 0 to 1, with higher values indicating more significant improvement effects. The Worse coefficient measures how much unfulfilled requirements negatively impact overall satisfaction, ranging from −1 to 0, with larger absolute values indicating more severe negative impacts. The formulas for satisfaction Si, dissatisfaction Di, and reverse requirement coefficient SR are as follows:


(2) $$\text{Better coefficient}\;Si=\frac{A+O}{A+O+M+I}$$

(3) $$\text{Worse coefficient Di}=\frac{-\;\left(O+M\right)}{A+O+M+I}$$

**Figure 5:**
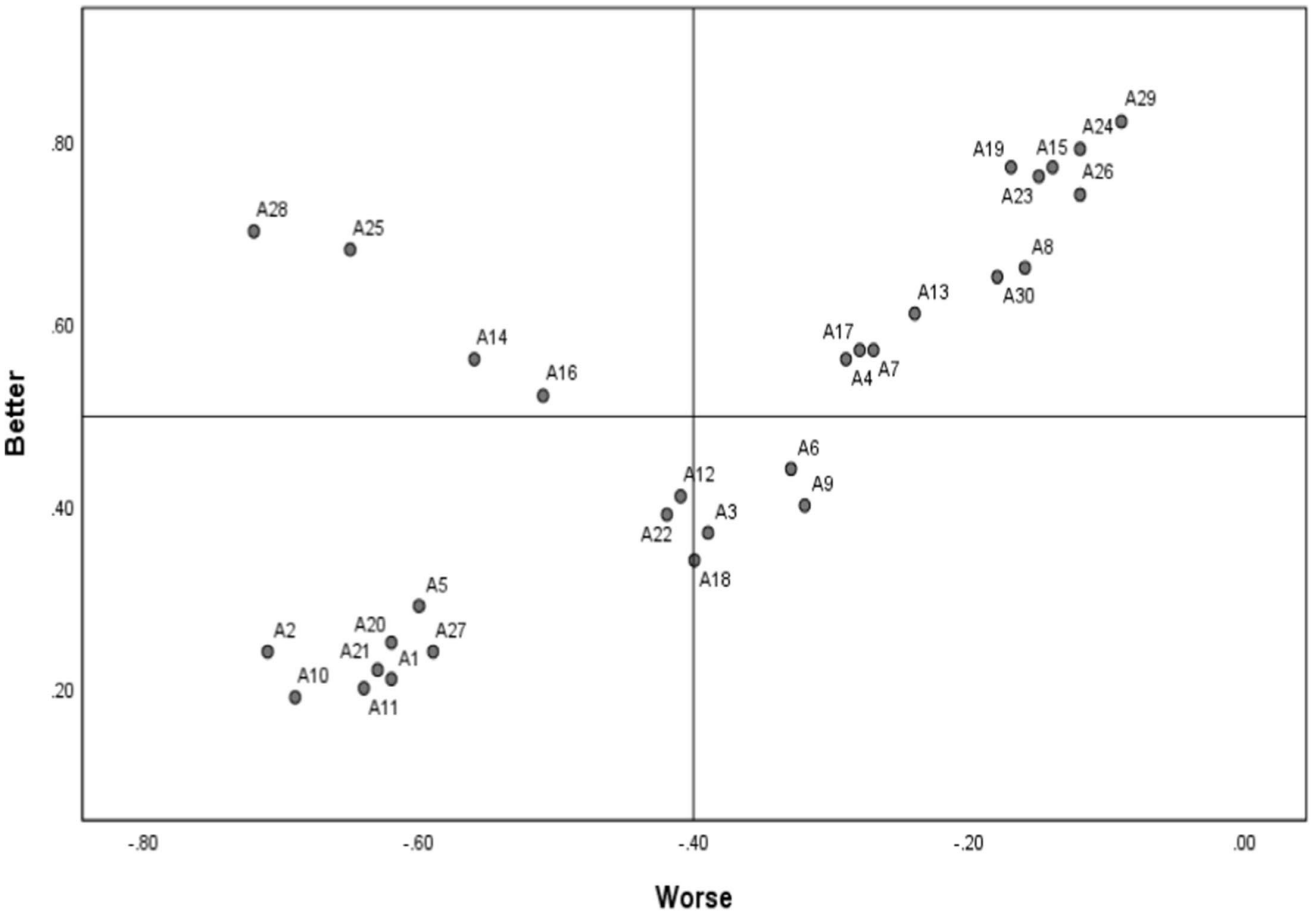
Better-worse coefficient analysis visualization.

By applying the aforementioned formulas to the questionnaire data, the importance and ranking of all user needs were determined in the following way (see Table 9 and Figure 5).

**Table 9:** Better-worse coefficient analysis results.

Functions/services	Better	Worse
A1	20.54%	−62.16%
A2	23.78%	−70.81%
A3	36.75%	−38.55%
A4	56.45%	−29.03%
A5	29.26%	−59.57%
A6	44.07%	−33.33%
A7	57.22%	−26.67%
A8	66.15%	−15.63%
A9	39.66%	−31.61%
A10	19.05%	−68.78%
A11	19.57%	−64.13%
A12	41.04%	−41.04%
A13	60.75%	−24.19%
A14	56.11%	−55.56%
A15	77.04%	−14.29%
A16	51.65%	−51.10%
A17	56.52%	−27.72%
A18	33.72%	−39.53%
A19	76.84%	−17.37%
A20	25.27%	−62.37%
A21	22.28%	−63.04%
A22	38.82%	−42.35%
A23	76.29%	−14.95%
A24	78.76%	−11.92%
A25	67.71%	−64.58%
A26	74.48%	−11.98%
A27	24.19%	−58.60%
A28	69.89%	−71.51%
A29	81.58%	−9.47%
A30	64.74%	−18.42%

From the above values, we observe significant differences in older adults’ preferences and needs regarding digital and social media usage. Most older adults have high expectations for customized platform designs (large fonts, simple layouts) and dedicated older adult modes, but these features are not widespread, resulting in higher “Worse” than “Better” ratios.

Older adults face major challenges in: Adapting to digital lifestyle, Mastering digital skills, and Distinguishing authentic online information. These areas show notably high “Worse” ratios.

Higher “Better” ratios appear in: Digital entertainment, Understanding younger generations’ lifestyles through digital means, and Digital community participation.

They also show expectations for: Personalized entertainment and learning content recommendations, Building digital social relationships and Participating in digital cultural activities.

Strong awareness exists regarding cybersecurity and privacy protection, with high demand for security knowledge and personal privacy protection. They also desire aesthetically pleasing designs with good functionality-operation matching.

In conclusion, older adults’ digital media usage presents both challenges and opportunities. Digital products and social media platforms need to focus on: Customized design, Accessible digital skill learning methods, Enhanced security and privacy features, and Rich digital cultural experiences addressing entertainment and social needs.

## 5 Discussion

### 5.1 Multidimensional challenges in digital cultural adaptation

The KANO model analysis reveals older adults’ needs for digital cultural services demonstrate hierarchical characteristics. Must-be requirements primarily include age-friendly design of digital media (47.80%), dedicated older adult modes (54.15%), and suitable learning methods (44.88%), indicating older adult users prioritize basic functional accessibility. This aligns with Amann-Hechenberger et al.’s (46) findings about interface barriers.

Attractive requirements manifest in understanding and participating in emerging digital culture, such as digital security (67.32%), social expression (66.34%), and entertainment methods (66.83%). Better-Worse analysis shows these needs significantly impact older adults’ satisfaction, echoing He et al.’s (18) research on digital participation promoting social integration.

One-dimensional requirements focus on social participation and skill mastery, including volunteer service participation (36.10%) and basic skill learning (50.73%). Their Better-Worse coefficients indicate these are key factors in improving older adults’ digital cultural adaptation. These findings provide clear guidance for policy-making: must-be requirements should become mandatory standards for digital products; attractive features should be encouraged through government incentive programs.

### 5.2 Cultural value differences and adaptation mechanisms

Intergenerational cultural value differences mainly reflect in perceptions of emerging economic forms and interaction methods. Older adults tend to be conservative toward internet celebrity economy, streamers, content creators, and virtual consumption behaviors. This value difference impacts intergenerational interactions: older adults show lower willingness to share digital content and rarely participate in younger generations’ digital entertainment activities.

While extensive research exists on intergenerational communication, these studies have predominantly focused on immigrant families, different ethnic groups, geographical regions, and occupational backgrounds, with limited attention to the digital cultural divide between generations within the same cultural background (47–51). This research gap is particularly significant in the context of China’s rapid digital transformation, where traditional values and emerging digital cultures create unique intergenerational dynamics.

KANO analysis further reveals significant generational differences in values. Indifferent requirements concentrate in understanding internet language (31.71%) and maintaining traditional values (32.20%), indicating older adults remain cautious about these emerging cultural elements. Better-Worse analysis shows meeting these needs has limited impact on overall satisfaction.

### 5.3 Digital inclusion and social support

Research shows enhancing older adults’ digital cultural adaptation requires multidimensional support. Digital infrastructure needs improvement, especially regarding urban–rural disparities. The “54th Statistical Report on China’s Internet Development” (2) shows urban internet users account for 72.1%, indicating room for rural digital penetration improvement.

Current digital skills training systems show clear inadequacies. As research (7) notes, few policies address specialized talent training and older adults’ digital skill training paths. This lack of training support may intensify learning anxiety. Meanwhile, necessary human services and assistance should be maintained, avoiding complete reliance on digital solutions.

Better-Worse coefficient analysis reveals hierarchical characteristics in older adults’ digital service needs. Lack of basic functions leads to high dissatisfaction (Worse coefficients generally above −60%), while innovative features significantly increase satisfaction (Better coefficients up to 81.58%). This suggests digital inclusion strategies should be implemented gradually, ensuring basic service accessibility while actively developing innovative features to enhance user experience.

Digital literacy training should adopt a tiered approach: basic operations first, then social participation, and finally innovative experiences. Family support satisfaction is low (2.054) yet highly influential, suggesting policymakers should design ‘digital companionship’ programs encouraging young family members to participate in older adults’ digital learning, such as offering volunteer credits and establishing intergenerational mentoring mechanisms.

### 5.4 Future development trends and policy recommendations

Based on KANO model analysis results and research findings, enhancing digital cultural services for the older adults requires comprehensive technological and policy support. Key technical improvements should focus on age-friendly design in interface interaction and functionality configuration, aligning with Song and Gu’s (8) findings on older adults’ preferences for simple operations. Content services need development to meet older adults’ spiritual and cultural needs (22), while learning support systems should center on family-based networks with community supplements, reflecting Chen et al.’s (17) emphasis on digital technology’s positive impact on older adults’ health and social participation.

Policy recommendations: (1) Establish national standards for older adult-friendly digital interfaces; (2) Develop digital adaptation assessment systems; (3) Incorporate digital cognitive education into older adults’ university curricula; and (4) Set up special funds to support innovative services.

Technical development recommendations: (1) Use the KANO model to evaluate feature priorities; (2) Design interfaces adaptable to different literacy levels; (3) Strengthen security features; and (4) Incorporate family assistance functions.

Policy support should be strengthened in several areas: promoting digital inclusion and cultural service system development (7), enhancing digital cultural cognitive education to help older adults understand emerging cultural expressions, and developing tools for intergenerational communication. Essential human services should be maintained alongside digital solutions. Based on KANO model results, policy priorities include standardizing age-friendly modifications for must-be requirements, encouraging innovative service development for attractive requirements, improving digital skills training systems for one-dimensional requirements, and recognizing the potential value of indifferent requirements in providing cultural identity for older adults.

### 5.5 Research limitations

This study collected data through online questionnaires, which means that respondents already have a certain level of digital access. This approach introduces several limitations: recall bias may affect older adult respondents’ ability to accurately report their digital usage patterns; self-assessment of digital skills may not reflect actual abilities; and the KANO model’s paired positive–negative questions may present comprehension challenges despite our clear instructions. Additionally, while family assistance with questionnaire completion enabled participation from less digitally confident seniors, it might have influenced responses.

We acknowledge that our online methodology inevitably excluded older adult individuals completely disconnected from digital technologies. Future research should expand the sample size to include more older adult people who lack access to digital devices, potentially employing mixed methods combining online and offline approaches to capture experiences across the digital divide spectrum.

These findings have significant theoretical and practical implications for advancing older adults’ digital inclusion and provide scientific basis for policy development. Future research could explore long-term effects of older adults’ digital cultural adaptation and its impact on quality of life.

## 6 Conclusion

This study analyzes the digital cultural adaptation needs of Chinese older adults using the KANO model, revealing their hierarchical characteristics. Basic needs emphasize age-friendly digital product design, with must-be requirements satisfaction rates of 40–55%; advanced needs show attractive requirements like digital security and social expression reaching 60–70%, indicating high expectations for digital life quality improvement.

The older adults face multiple adaptation challenges including cognitive barriers in understanding new digital expressions and skill gaps in device usage. A significant urban–rural digital divide exists, with urban users at 72.1%. Better-Worse analysis indicates basic function absence leads to over 60% dissatisfaction, while innovative features can increase satisfaction to 81.58%.

Based on these findings, policymakers should: (1) incorporate older adult-friendly design standards into mandatory digital product regulations; (2) establish tiered digital training systems adapted to different group needs; and (3) support incentive mechanisms for family involvement in older adults’ digital learning. Technology developers should: (1) prioritize ensuring basic functionality usability; (2) develop adaptive interfaces considering cognitive level differences; and (3) enhance security features, particularly focusing on privacy protection.

Through comprehensive application of technical support, content services, and policy measures, older adults’ digital cultural adaptation capability can be significantly improved, promoting intergenerational cultural integration and achieving better integration of older adults into digital society.

## Data Availability

The raw data collected in this study contains sensitive personal information from elderly participants and is subject to strict privacy protections under our institutional ethics protocol (IRB Number: BNU202411270217). To protect participant confidentiality and comply with ethical guidelines regarding vulnerable populations, the original dataset will not be shared publicly or with third parties.
